# Supplementary material for: Microbial community structure and microbial networks correspond to nutrient gradients within coastal wetlands of the Laurentian Great Lakes
Source: FEMS Microbiol Ecol. 2019 Mar 11;95(4):fiz033. doi: 10.1093/femsec/fiz033 (PMC6447756; doi:10.1093/femsec/fiz033)
Supplement: Supplemental Files [file fiz033_supplemental_files.zip › Supplementary_Material_figures.docx]

## Supplementary Figures

**
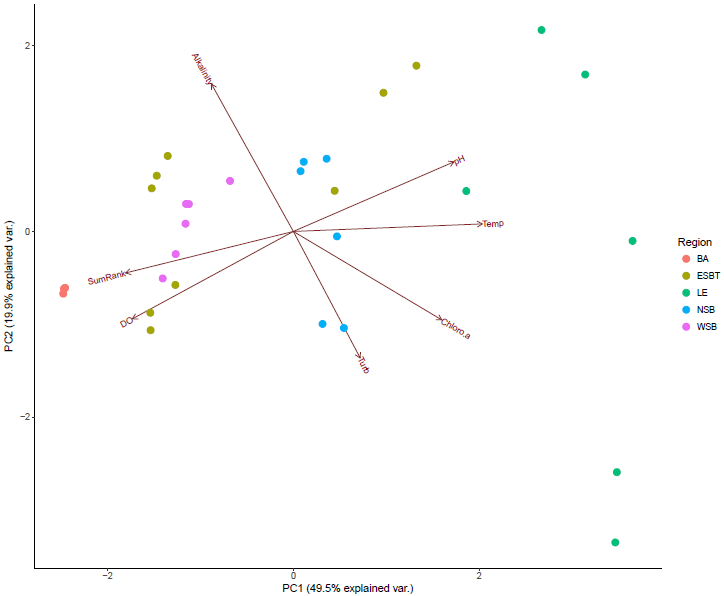
**

**Supplemental Figure 1.** Principal Components Analysis demonstrating separation of coastal wetland sampling locations by site water quality data. Points are color-coded by region. Percentages on axes represent explained variance of that principal component. Vectors represent impact of specific environmental variables on sample distribution. Choloro.a = chlorophyll A, DO = dissolved oxygen, Temp = water temperature, Turb = water turbidity. SumRank represents SumRank values calculated for each wetland site as outlined by Uzarski *et al.* (2017). DO also represents redox potential values, which correlated significantly (r > 0.7, p ≤ .001). Temp also represents conductivity and total dissolved solids values, which correlated significantly (r > 0.7, p ≤ .001).


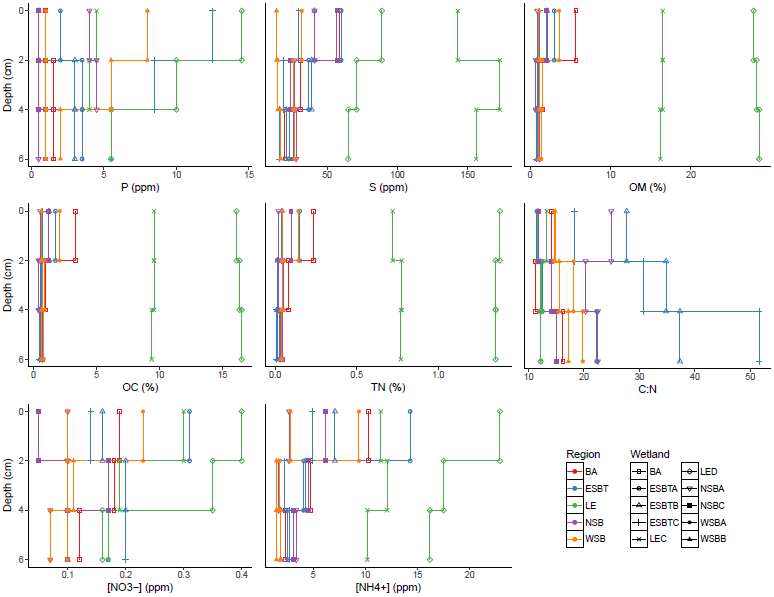


**Supplemental Figure 2.** Depth profiles demonstrating trends in measured environmental variables with increasing depth among wetland regions and sites. Colors represent wetland regions, whereas point shapes represent distinct wetland sites.


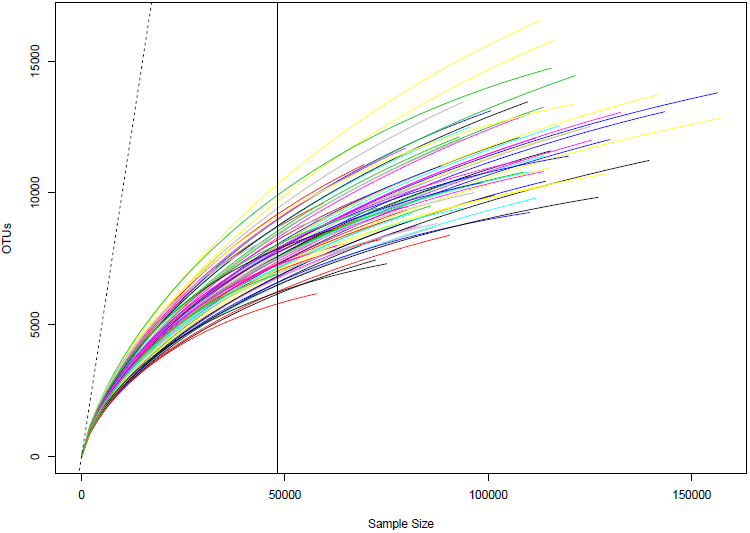


**Supplemental Figure 3.** Rarefaction curve analysis demonstrating sequencing depth for alpha diversity analyses. Different colored lines represent different samples. The vertical black line represents the sequencing depth used to standardize all samples for alpha diversity analysis. The dashed line represents the 1:1 slope.


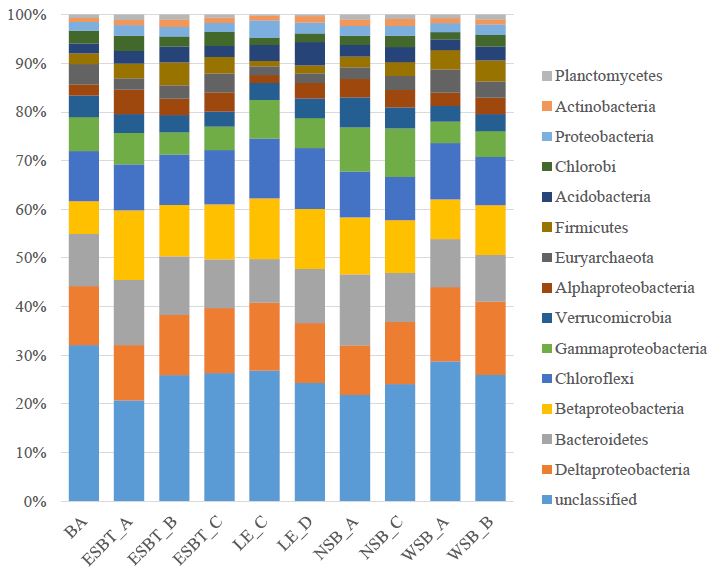


**Supplemental Figure 4.** Phylogenetic composition of each wetland site at the level of Phylum. Displayed phyla composed at least 1% of relative abundance within at least one wetland site.


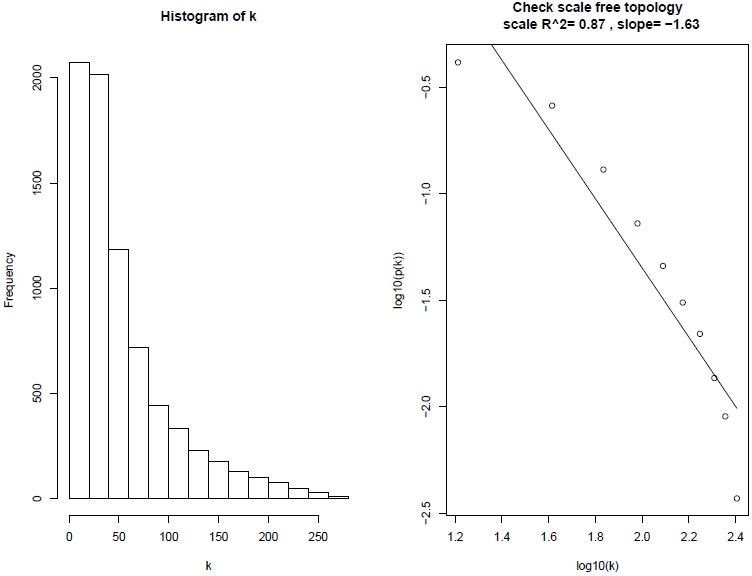


**Supplemental Figure 5.** Plots demonstrating regression of the frequency distribution of node connectivity against node connectivity.


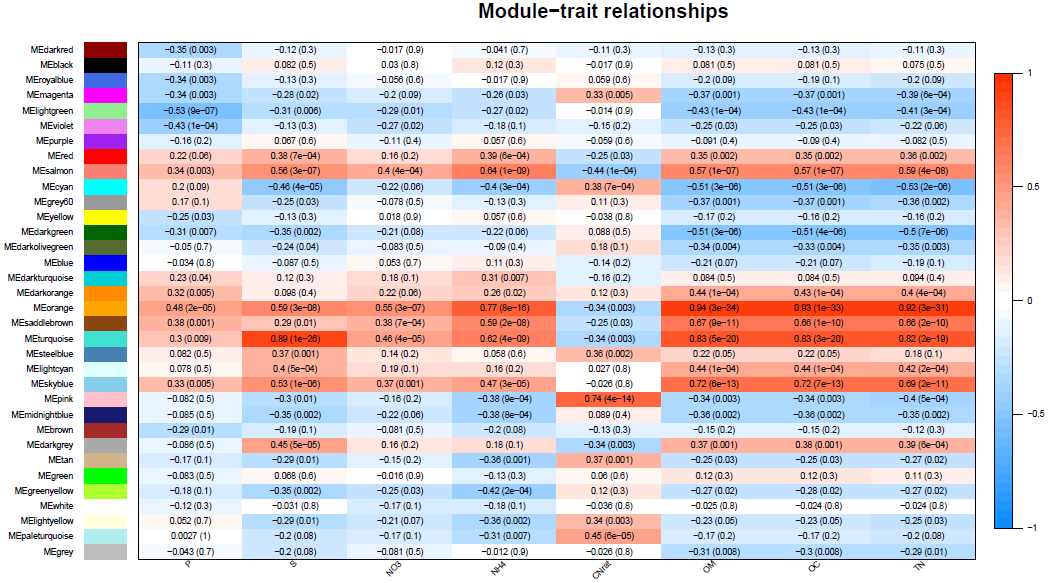


**Supplemental Figure 6.** Individual correlations of established subnetworks with environmental parameters.


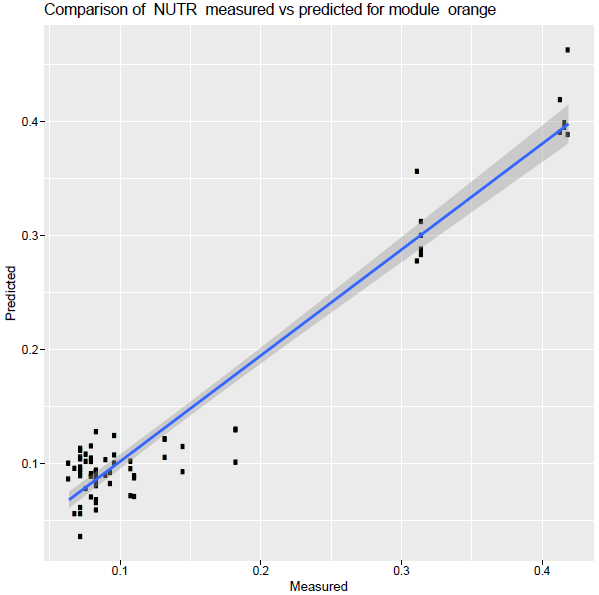


**Supplemental Figure 7.** Partial least squares analysis results predicting OC (NUTR) values using relative abundance values of OTUs within the module most connected to OC (NUTR).


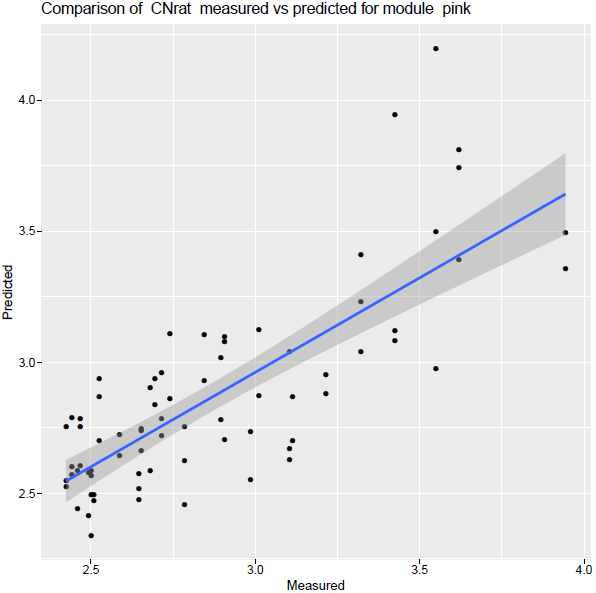


**Supplemental Figure 8.** Partial least squares analysis results predicting C:N values using relative abundance values of OTUs within the module most connected to C:N.
