# Supplementary material for: Microbial community structure and microbial networks correspond to nutrient gradients within coastal wetlands of the Laurentian Great Lakes
Source: FEMS Microbiol Ecol. 2019 Mar 11;95(4):fiz033. doi: 10.1093/femsec/fiz033 (PMC6447756; doi:10.1093/femsec/fiz033)
Supplement: Supplemental Files [file fiz033_supplemental_files.zip › 10_29_Supplementary_Material_tables.docx]

## Supplementary Tables

Supplemental Table 1. Average pH associated with overlying water of each wetland region. Avg pH = average pH, STDev = standard deviation, n = number of replicate readings taken per region.

|  | Avg pH | STDev | n |
| --- | --- | --- | --- |
| BA | 8.31 | 0.01 | 3 |
| ESBT | 8.15 | 0.40 | 9 |
| LE | 7.26 | 0.24 | 6 |
| NSB | 7.72 | 0.16 | 6 |
| WSB | 8.39 | 0.26 | 6 |

| Supplemental Table 2. Soil physical and chemical data corresponding to each wetland site. | | | | | | | |  |  |  |  |  |
| --- | --- | --- | --- | --- | --- | --- | --- | --- | --- | --- | --- | --- |
|  |  |  |  |  |  |  |  |  |  |  |  |  |
| wetland | region | depth | pH | brayP.(ppm) | sulfur.(ppm) | organic matter.(%) | organic carbon.(%) | tn.(%) | c:n | NO3.(ppm) | NH4.(ppm) | |
| BA | BA | 1 | 7.9 | 1 | 59 | 5.7 | 3.3 | 0.234 | 14.1 | 0.19 | 10.3 |  |
| BA | BA | 2 | 8.3 | 1.5 | 31 | 1.6 | 0.928074 | 0.082 | 11.3 | 0.18 | 4.8 |  |
| BA | BA | 3 | 8.5 | 1.5 | 20 | 1.2 | 0.696056 | 0.043 | 16.2 | 0.12 | 2.4 |  |
| ESBTA | ESBT | 1 | 8 | 2 | 60 | 3 | 1.740139 | 0.151 | 11.5 | 0.31 | 14.3 |  |
| ESBTA | ESBT | 2 | 8.3 | 3.5 | 37 | 1.1 | 0.638051 | 0.051 | 12.5 | 0.1 | 4.1 |  |
| ESBTA | ESBT | 3 | 8.4 | 3.5 | 23 | 1 | 0.580046 | 0.026 | 22.3 | 0.17 | 2.6 |  |
| ESBTB | ESBT | 1 | 8.2 | 0.5 | 41 | 2.1 | 1.218097 | 0.044 | 27.7 | 0.16 | 7.1 |  |
| ESBTB | ESBT | 2 | 8.5 | 3 | 39 | 0.9 | 0.522042 | 0.015 | 34.8 | 0.2 | 4.3 |  |
| ESBTB | ESBT | 3 | 8.5 | 3 | 21 | 0.9 | 0.522042 | 0.014 | 37.3 | 0.1 | 1.9 |  |
| ESBTC | ESBT | 1 | 8.1 | 12.5 | 30 | 1.2 | 0.696056 | 0.038 | 18.3 | 0.14 | 4.9 |  |
| ESBTC | ESBT | 2 | 8.3 | 8.5 | 19 | 0.9 | 0.522042 | 0.017 | 30.7 | 0.1 | 2.3 |  |
| ESBTC | ESBT | 3 | 8.4 | 5.5 | 16 | 0.8 | 0.464037 | 0.009 | 51.6 | 0.2 | 2.7 |  |
| LEC | LE | 1 | 7.8 | 4.5 | 143 | 16.5 | 9.570766 | 0.721 | 13.3 | 0.3 | 11.5 |  |
| LEC | LE | 2 | 7.7 | 4 | 173 | 16.5 | 9.570766 | 0.777 | 12.3 | 0.19 | 12.1 |  |
| LEC | LE | 3 | 7.7 | 5.5 | 156 | 16.2 | 9.396752 | 0.771 | 12.2 | 0.17 | 10.2 |  |
| LED | LE | 1 | 6.6 | 14.5 | 89 | 27.8 | 16.12529 | 1.378 | 11.7 | 0.4 | 22.9 |  |
| LED | LE | 2 | 6.4 | 10 | 71 | 28.2 | 16.35731 | 1.351 | 12.1 | 0.35 | 17.5 |  |
| LED | LE | 3 | 6.3 | 5.5 | 65 | 28.5 | 16.53132 | 1.352 | 12.2 | 0.16 | 16.2 |  |
| NSBA | NSB | 1 | 8.2 | 4 | 41 | 0.9 | 0.522042 | 0.021 | 24.9 | 0.1 | 2.7 |  |
| NSBA | NSB | 2 | 8.4 | 4.5 | 27 | 0.7 | 0.406032 | 0.02 | 20.3 | 0.1 | 1.7 |  |
| NSBA | NSB | 3 | 8.2 | 0.5 | 28 | 1.2 | 0.696056 | 0.031 | 22.5 | 0.07 | 3.4 |  |
| NSBC | NSB | 1 | 8.1 | 0.5 | 57 | 2 | 1.160093 | 0.098 | 11.8 | 0.05 | 6.2 |  |
| NSBC | NSB | 2 | 8.3 | 0.5 | 24 | 1.2 | 0.696056 | 0.049 | 14.2 | 0.17 | 4.6 |  |
| NSBC | NSB | 3 | 8.3 | 1 | 26 | 1.2 | 0.696056 | 0.046 | 15.1 | 0.1 | 3.1 |  |
| WSBA | WSB | 1 | 8.1 | 1 | 32 | 3.6 | 2.088167 | 0.143 | 14.6 | 0.23 | 9.4 |  |
| WSBA | WSB | 2 | 8.4 | 1 | 26 | 1.5 | 0.87007 | 0.048 | 18.1 | 0.1 | 1.8 |  |
| WSBA | WSB | 3 | 8.3 | 1 | 27 | 1.4 | 0.812065 | 0.041 | 19.8 | 0.1 | 1.9 |  |
| WSBB | WSB | 1 | 8.3 | 8 | 14 | 1.1 | 0.638051 | 0.043 | 14.8 | 0.1 | 2.8 |  |
| WSBB | WSB | 2 | 8.3 | 5.5 | 15 | 1.2 | 0.696056 | 0.045 | 15.5 | 0.11 | 1.5 |  |
| WSBB | WSB | 3 | 8.4 | 2 | 17 | 1.1 | 0.638051 | 0.037 | 17.2 | 0.07 | 1.5 |  |
|  |  |  |  |  |  |  |  |  |  |  |  |  |

| Supplemental Table 3. Environmental measurements taken with a YSI multiprobe at each core collection location. Rep = replicate, DO = dissolved oxygen, Temp = temperature, Sp Cond. = specific conductivity, TDS = total dissolved solids, Redox = redox potential, Chloro.a = Chlorophyll-A, Total Alk = total alkalinity. Total Alkalinity was only measured at one replicate core location per site. | | | | | | | | | | | | | | | | |
| --- | --- | --- | --- | --- | --- | --- | --- | --- | --- | --- | --- | --- | --- | --- | --- | --- |
| **Location** | **Date** | **Rep** | **Core Title** | **pH** | **Vegitation Zone** | **Lat/Long** | **DO (mg/L)** | **DO % Saturation** | **Temp** | **Sp Cond.** | **TDS** | **Turbidity** | **Redox** | **Chl.a** | **Total Alk** |  |
|  |  |  |  |  |  |  |  |  |  |  |  |  |  |  |  |  |
| Beaver Archipelago | Jul/21-25/2014 | 1 | BA_1 | 8.32 | Inner Schoeno | N45.79785/W085.50790 | 9.44 | 103.2 | 19.958 | 275.4 | 179 | 0.35 | 185.1 | 0.01 | 107 |  |
|  |  | 2 | BA_2 | 8.3 | Inner Schoeno | N45.79797/W085.50802 | 9.08 | 99.5 | 19.641 | 276.9 | 180 | 0.3 | 182.8 | 0.09 | N/A |  |
|  |  | 3 | BA_3 | 8.3 | Inner Schoeno | N45.79803/W085.50836 | 9 | 97.9 | 19.42 | 280 | 182 | 0.34 | 181.1 | 0.19 | N/A |  |
| East Saginaw Bay/Thumb | Jun/30-Jul/3/2014 | 1 | ESBT_A_1 | 7.71 | Typha | N43.59804/W083.66421 | 7.04 | 86.7 | 25.96 | 487.4 | 317 | -1.51 | 122.2 | 5.13 | 181.5 |  |
|  |  | 2 | ESBT_A_2 | 7.72 | Typha | N43.59686/W083.66687 | 5.45 | 70.2 | 26.2 | 491.8 | 320 | -1.7 | 124.6 | 5.3 | N/A |  |
|  |  | 3 | ESBT_A_3 | 8.51 | Typha | N43.59696/W083.66791 | 9.47 | 116.9 | 25.87 | 434.4 | 282 | 0.05 | 106.9 | 6.1 | N/A |  |
| East Saginaw Bay/Thumb | Jun/30-Jul/3/2014 | 1 | ESBT_B_1 | 8.29 | Inner Schoeno | N43.80492/W083.45908 | 7.42 | 82.9 | 20.69 | 352.2 | 229 | 0.27 | 217.8 | 2.77 | 99 |  |
|  |  | 2 | ESBT_B_2 | 8.66 | Inner Schoeno | N43.80444/W083.45920 | 8.5 | 94.8 | 20.76 | 350.5 | 228 | 0.95 | 209.4 | 2.09 | N/A |  |
|  |  | 3 | ESBT_B_3 | 8.8 | Inner Schoeno | N43.80401/W083.45968 | 8.86 | 99.3 | 20.921 | 346.9 | 225 | 1.41 | 202.6 | 2.24 | N/A |  |
| East Saginaw Bay/Thumb | Jun/30-Jul/3/2014 | 1 | ESBT_C_1 | 7.79 | Inner Schoeno | N43.88379/W083.33350 | 7.72 | 89.1 | 22.27 | 387.8 | 252 | -2.31 | 203.1 | 1.55 | 139 |  |
|  |  | 2 | ESBT_C_2 | 7.89 | Inner Schoeno | N43.88329/W083.33371 | 8.44 | 97.7 | 22.53 | 386.2 | 251 | -2.11 | 195.6 | 1.66 | N/A |  |
|  |  | 3 | ESBT_C_3 | 8 | Inner Schoeno | N43.88282/W083.33437 | 8.89 | 103.3 | 22.86 | 383 | 249 | -2.01 | 115.6 | 1.75 | N/A |  |
| Lake Erie | Jun/16-20/2014 | 1 | LE_C_1 | 7.7 | Typha | N41.54284/W083.21847 | 7.77 | 100 | 28.3 | 486 | 316 | 2.52 | 161.4 | 17.61 | 12.2 |  |
|  |  | 2 | LE_C_2 | 7.11 | Typha | N41.90491/W083.36430 | 3.15 | 40 | 27 | 459 | 299 | -0.87 | 121.5 | 7.56 | N/A |  |
|  |  | 3 | LE_C_3 | 7.35 | Typha | N41.54307/W083.21869 | 6.22 | 80.9 | 28.4 | 444.6 | 289 | 5.24 | 128.5 | 9.51 | N/A |  |
| Lake Erie | Jun/16-20/2014 | 1 | LE_D_1 | 7.1 | Typha | N41.91526/W083.34155 | 1.13 | 14.6 | 25.765 | 370.8 | 241 | -1.83 | 39.3 | 3.65 | 110 |  |
|  |  | 2 | LE_D_2 | 7.36 | Typha | N41.91547/W083.34190 | 4.4 | 55.8 | 26.545 | 367.1 | 239 | 1.68 | 95.7 | 3.5 | N/A |  |
|  |  | 3 | LE_D_3 | 6.96 | Typha | N41.91546/W083.34245 | 2.27 | 27.9 | 25.668 | 378.4 | 246 | 0.68 | 69.9 | 3.95 | N/A |  |
| Northwest Saginaw Bay | Jul/7-11/2014 | 1 | NSB_A_1 | 7.98 | Typha | N43.91784/W083.89832 | 6.93 | 83.7 | 24.967 | 347.2 | 226 | 5.19 | 171.9 | 1.6 | 116.5 |  |
|  |  | 2 | NSB_A_2 | 7.87 | Typha | N43.91943/W083.89661 | 6.8 | 83.1 | 25.45 | 465.8 | 303 | 5.46 | 154.1 | 1.99 | N/A |  |
|  |  | 3 | NSB_A_3 | 7.72 | Typha | N43.92023/W083.89693 | 5.65 | 68.8 | 24.946 | 442.7 | 288 | 2.57 | 159.2 | 1.38 | N/A |  |
| Northwest Saginaw Bay | Jul/7-11/2014 | 1 | NSB_C_1 | 7.59 | Typha | N43.98619/W083.79672 | 6.63 | 81.5 | 25.638 | 396.3 | 258 | 1.16 | 136.1 | 1.74 | 162.5 |  |
|  |  | 2 | NSB_C_2 | 7.58 | Typha | N43.98563/W083.79732 | 6.94 | 85.8 | 25.643 | 390.4 | 254 | 1.41 | 138.6 | 1.71 | N/A |  |
|  |  | 3 | NSB_C_3 | 7.56 | Typha | N43.98544/W083.79789 | 6.08 | 75.4 | 26.114 | 393.3 | 256 | 1.58 | 143.2 | 1.62 | N/A |  |
| West Saginaw Bay | Jun/23-27/2014 | 1 | WSB_A_1 | 7.92 | Inner Schoeno | N43.73919/W083.94518 | 6.94 | 80.2 | 22.25 | 424.4 | 276 | -2.04 | 157.6 | 3.39 | 124 |  |
|  |  | 2 | WSB_A_2 | 8.41 | Inner Schoeno | N43.73944/W083.94510 | 8.16 | 93.8 | 22.15 | 373.2 | 243 | -1.84 | 166.5 | 2.5 | N/A |  |
|  |  | 3 | WSB_A_3 | 8.47 | Inner Schoeno | N43.73976/W083.94541 | 8.18 | 94.4 | 22.26 | 366.4 | 238 | -2 | 169.9 | 2.8 | N/A |  |
| West Saginaw Bay | Jun/23-27/2014 | 1 | WSB_B_1 | 8.27 | Inner Schoeno | N43.45223/W083.56280 | 8.1 | 93.8 | 22.36 | 367 | 239 | -1.48 | 178.7 | 2.48 | 116.5 |  |
|  |  | 2 | WSB_B_2 | 8.48 | Inner Schoeno | N43.45202/W083.56272 | 8.68 | 99.7 | 22.32 | 358 | 233 | -0.47 | 176.1 | 2.21 | N/A |  |
|  |  | 3 | WSB_B_3 | 8.79 | Inner Schoeno | N43.45155/W083.56223 | 9.66 | 110.6 | 22.3 | 331 | 216 | -0.47 | 170.1 | 2.95 | N/A |  |

Supplemental Table 4. LME and ANOVA results on influence of depth and region on significant differences in Chao1 estimates. Significance codes: 0 '***' 0.001 '**' 0.01 '*' 0.05.

|  | | | | numDF | | | | denDF | | | F-value | | | p-value | | | |  | | |  | | |  | | |  | |  |  | |  |
| --- | --- | --- | --- | --- | --- | --- | --- | --- | --- | --- | --- | --- | --- | --- | --- | --- | --- | --- | --- | --- | --- | --- | --- | --- | --- | --- | --- | --- | --- | --- | --- | --- |
| (Intercept) | | | | 1 | | | | 54 | | | 820.3903 | | | <.0001 | | | |  | | |  | | |  | | |  | |  |  | |  |
| Depth | | | | 2 | | | | 54 | | | 2.1482 | | | 0.1266 | | | |  | | |  | | |  | | |  | |  |  | |  |
| Region | | | | 4 | | | | 5 | | | 8.38 | | | 0.0193 | | | | * | | |  | | |  | | |  | |  |  | |  |
| Depth:Region | | | | 8 | | | | 54 | | | 0.7243 | | | 0.6693 | | | |  | | |  | | |  | | |  | |  |  | |  |
|  | | | |  | | | |  | | |  | | |  | | | |  | | |  | | |  | | |  | |  |  | |  |
| Pairwise comparisons with bonferroni adjustments of Chao1 estimates between regions. Significance codes: 0 '***' 0.001 '**' 0.01 '*' 0.05. | | | | | | | | | | | | | | | | | | | | | | | | | | | | | | |  | |
|  | | | | BA | | | | ESBT | | | LE | | | NSB | | | | WSB | | |  | | |  | | |  | |  |  | |  |
| BA | | | | - | | | |  | | |  | | |  | | | |  | | |  | | |  | | |  | |  |  | |  |
| ESBT | | | | *n.s.* | | | | - | | |  | | |  | | | |  | | |  | | |  | | |  | |  |  | |  |
| LE | | | | *n.s.* | | | | * | | | - | | |  | | | |  | | |  | | |  | | |  | |  |  | |  |
| NSB | | | | *n.s.* | | | | *n.s.* | | | *** | | | - | | | |  | | |  | | |  | | |  | |  |  | |  |
| WSB | | | | * | | | | * | | | *** | | | *n.s.* | | | | - | | |  | | |  | | |  | |  |  | |  |
|  | | | |  | | | |  | | |  | | |  | | | |  | | |  | | |  | | |  | |  |  | |  |
|  | | | |  | | | |  | | |  | | |  | | | |  | | |  | | |  | | |  | |  |  | |  |
| LM and ANOVA results on influence of depth and wetland on significant differences in Chao1 estimates. | | | | | | | | | | | | | | | | | | | | | | | | | | | | | | | | |
|  | | | | Df | | | | Sum Sq | | | Mean Sq | | | F value | | | | Pr(>F) | | |  | | |  | | |  | |  |  | |  |
| Wetland | | | | 9 | | | | 677208771 | | | 75245419 | | | 16.7752 | | | | 1.54E-11 | | | *** | | |  | | |  | |  |  | |  |
| Depth | | | | 2 | | | | 17147004 | | | 8573502 | | | 1.9114 | | | | 0.16 | | |  | | |  | | |  | |  |  | |  |
| Wetland:Depth | | | | 18 | | | | 41069808 | | | 2281656 | | | 0.5087 | | | | 0.9395 | | |  | | |  | | |  | |  |  | |  |
| Residuals | | | | 44 | | | | 197362151 | | | 4485503 | | |  | | | |  | | |  | | |  | | |  | |  |  | |  |
|  | | | |  | | | |  | | |  | | |  | | | |  | | |  | | |  | | |  | |  |  | |  |
|  | | | | | | | | | | | | | | | | | | | | | | | | | | | | | | |  | |
|  | | BA_A | | | ESBT_A | | ESBT_B | | | ESBT_C | | | LE_C | | LE_D | | NSB_A | | | NSB_C | | | WSB_A | | | WSB_B |  |  |  |  |  |  |
| BA_A | | - | | |  | |  | | |  | | |  | |  | |  | | |  | | |  | | |  |  |  |  |  |  |  |
| ESBT_A | | * | | | - | |  | | |  | | |  | |  | |  | | |  | | |  | | |  |  |  |  |  |  |  |
| ESBT_B | | *n.s.* | | | ** | | - | | |  | | |  | |  | |  | | |  | | |  | | |  |  |  |  |  |  |  |
| ESBT_C | | *n.s.* | | | *n.s.* | | *n.s.* | | | - | | |  | |  | |  | | |  | | |  | | |  |  |  |  |  |  |  |
| LE_C | | *n.s.* | | | *** | | *n.s.* | | | * | | | - | |  | |  | | |  | | |  | | |  |  |  |  |  |  |  |
| LE_D | | *n.s.* | | | *** | | *n.s.* | | | * | | | *n.s.* | | - | |  | | |  | | |  | | |  |  |  |  |  |  |  |
| NSB_A | | *n.s.* | | | *n.s.* | | *n.s.* | | | *n.s.* | | | ** | | *** | | - | | |  | | |  | | |  |  |  |  |  |  |  |
| NSB_C | | *** | | | *n.s.* | | *** | | | ** | | | *** | | *** | | *n.s.* | | | - | | |  | | |  |  |  |  |  |  |  |
| WSB_A | | *** | | | *n.s.* | | *** | | | * | | | *** | | *** | | *n.s.* | | | *n.s.* | | | - | | |  |  |  |  |  |  |  |
| WSB_B | | *** | | | *n.s.* | | *** | | | *** | | | *** | | *** | | ** | | | *n.s.* | | | *n.s.* | | | - |  |  |  |  |  |  |
|  | | | |  | | | |  | | |  | | |  | | | |  | | |  | | |  | | |  | |  |  | |  |
|  | | | |  | | | |  | | |  | | |  | | | |  | | |  | | |  | | |  | |  |  | |  |
| LME and ANOVA results on influence of depth and region on Shannon diversity levels. | | | | | | | | | | | | | | | | | | | | | | | | | | | | | | |  | |
|  | | | | numDF | | | | denDF | | | F-value | | | p-value | | | |  | | |  | | |  | | |  | |  |  | |  |
| (Intercept) | | | | 1 | | | | 54 | | | 49157.36 | | | <.0001 | | | |  | | |  | | |  | | |  | |  |  | |  |
| Depth | | | | 2 | | | | 54 | | | 1.69 | | | 0.1942 | | | |  | | |  | | |  | | |  | |  |  | |  |
| Region | | | | 4 | | | | 5 | | | 4.15 | | | 0.0752 | | | |  | | |  | | |  | | |  | |  |  | |  |
| Depth:Region | | | | 8 | | | | 54 | | | 0.36 | | | 0.9392 | | | |  | | |  | | |  | | |  | |  |  | |  |
|  | | | |  | | | |  | | |  | | |  | | | |  | | |  | | |  | | |  | |  |  | |  |
|  | | | |  | | | |  | | |  | | |  | | | |  | | |  | | |  | | |  | |  |  | |  |
| LM and ANOVA results on influence of wetland and region on Shannon diversity levels. | | | | | | | | | | | | | | | | | | | | | | | | | | | | | | |  | |
|  | | | | Df | | | | Sum Sq | | | Mean Sq F | | | value | | | | Pr(>F) | | |  | | |  | | |  | |  |  | |  |
| Wetland | | | | 9 | | | | 1.70012 0 | | | 0.188903 | | | 4.5746 0 | | | | 0.0002734 | | | *** | | |  | | |  | |  |  | |  |
| Depth | | | | 2 | | | | 0.14169 0 | | | 0.070845 | | | 1.7156 0 | | | | 0.1916649 | | |  | | |  | | |  | |  |  | |  |
| Wetland:Depth | | | | 18 | | | | 0.54100 0 | | | 0.030055 | | | 0.7278 0 | | | | 0.7647738 | | |  | | |  | | |  | |  |  | |  |
| Residuals | | | | 44 | | | | 1.81694 0 | | | 0.041294 | | |  | | | |  | | |  | | |  | | |  | |  |  | |  |
|  | | | |  | | | |  | | |  | | |  | | | |  | | |  | | |  | | |  | |  |  | |  |
| Pairwise comparisons with bonferroni adjustments of Shannon diversity estimates between wetland sites. | | | | | | | | | | | | | | | | | | | | | | | | | | | |  |  |  |  |  |
|  | BA_A | | ESBT_A | | | ESBT_B | | | ESBT_C | | | LE_C | | LE_D | | NSB_A | | | NSB_C | | | WSB_A | | | WSB_B | |  |  |  |  |  |  |
| BA_A | - | |  | | |  | | |  | | |  | |  | |  | | |  | | |  | | |  | |  |  |  |  |  |  |
| ESBT_A | *n.s.* | | - | | |  | | |  | | |  | |  | |  | | |  | | |  | | |  | |  |  |  |  |  |  |
| ESBT_B | *n.s.* | | *n.s.* | | | - | | |  | | |  | |  | |  | | |  | | |  | | |  | |  |  |  |  |  |  |
| ESBT_C | *n.s.* | | *n.s.* | | | *n.s.* | | | - | | |  | |  | |  | | |  | | |  | | |  | |  |  |  |  |  |  |
| LE_C | *n.s.* | | * | | | *n.s.* | | | *n.s.* | | | - | |  | |  | | |  | | |  | | |  | |  |  |  |  |  |  |
| LE_D | *n.s.* | | *** | | | *n.s.* | | | * | | | *n.s.* | | - | |  | | |  | | |  | | |  | |  |  |  |  |  |  |
| NSB_A | *n.s.* | | * | | | *n.s.* | | | *n.s.* | | | *n.s.* | | *n.s.* | | - | | |  | | |  | | |  | |  |  |  |  |  |  |
| NSB_C | *n.s.* | | *n.s.* | | | *n.s.* | | | *n.s.* | | | *n.s.* | | *n.s.* | | *n.s.* | | | - | | |  | | |  | |  |  |  |  |  |  |
| WSB_A | *n.s.* | | *n.s.* | | | *n.s.* | | | *n.s.* | | | *n.s.* | | *n.s.* | | *n.s.* | | | *n.s.* | | | - | | |  | |  |  |  |  |  |  |
| WSB_B | *n.s.* | | *n.s.* | | | *n.s.* | | | *n.s.* | | | *n.s.* | | ** | | *n.s.* | | | *n.s.* | | | *n.s.* | | | - | |  |  |  |  |  |  |

Supplemental Table 5. Environmental correlations with NDMS structure of all regions and NMDS structure of individual regions. Also reported are perMANOVA results when applicable testing the effect of wetland site and depth on microbial community structure within individual wetland regions. Significant codes correspond as following: 0 ‘***’ 0.001 ‘**’ 0.01 ‘*’ 0.05 ‘.’ 0.1 ‘ ’ 1.

| **All regions** |  |  |  |  |  |  |  |
| --- | --- | --- | --- | --- | --- | --- | --- |
| *envfit* correlations |  |  |  |  |  |  |  |
|  | NMDS1 | NMDS2 | r2 | Pr(>r) |  |  |  |
| depth | 0.96787 | -0.25144 | 0.4069 | 0.001 | *** |  |  |
| P | 0.19914 | 0.97997 | 0.1367 | 0.01 | ** |  |  |
| S | 0.26078 | 0.9654 | 0.4082 | 0.001 | *** |  |  |
| NO3 | 0.10152 | 0.99483 | 0.1989 | 0.001 | *** |  |  |
| CNrat | 0.32857 | -0.94448 | 0.1128 | 0.016 | * |  |  |
| NUTR | 0.32504 | 0.9457 | 0.6019 | 0.001 | *** |  |  |
|  |  |  |  |  |  |  |  |
| **BA** |  |  |  |  |  |  |  |
| *envfit* correlations |  |  |  |  |  |  |  |
|  | NMDS1 | NMDS2 | r2 | Pr(>r) |  |  |  |
| depth | 0.99883 | -0.04831 | 0.8263 | 0.012 | * |  |  |
| P | 0.98539 | -0.17029 | 0.7626 | 0.023 | * |  |  |
| S | -0.99446 | 0.10516 | 0.8525 | 0.008 | ** |  |  |
| NO3 | -0.99831 | -0.0581 | 0.6024 | 0.068 | . |  |  |
| CNrat | 0.7076 | 0.70661 | 0.0418 | 0.858 |  |  |  |
| NUTR | -0.98995 | 0.1414 | 0.816 | 0.009 | ** |  |  |
|  |  |  |  |  |  |  |  |
| perMANOVA |  |  |  |  |  |  |  |
|  | Df | SumsOfSqs | MeanSqs | F.Model | R2 | Pr(>F) |  |
| depth | 2 | 0.46796 | 0.23398 | 2.1205 | 0.41412 | 0.006 | ** |
| Residuals | 6 | 0.66205 | 0.11034 | 0.58588 |  |  |  |
| Total | 8 | 1.13001 | 1 |  |  |  |  |
|  |  |  |  |  |  |  |  |
| **ESBT** |  |  |  |  |  |  |  |
| *envfit* correlations |  |  |  |  |  |  |  |
|  | NMDS1 | NMDS2 | r2 | Pr(>r) |  |  |  |
| depth | 0.84565 | -0.53374 | 0.5242 | 0.001 | *** |  |  |
| P | 0.01263 | -0.99992 | 0.3506 | 0.008 | ** |  |  |
| S | -0.67377 | 0.73895 | 0.6053 | 0.001 | *** |  |  |
| NO3 | -0.92296 | 0.3849 | 0.1455 | 0.173 |  |  |  |
| CNrat | 0.8864 | 0.46291 | 0.6357 | 0.001 | *** |  |  |
| NUTR | -0.77573 | 0.63106 | 0.5112 | 0.001 | *** |  |  |
|  |  |  |  |  |  |  |  |
| perMANOVA |  |  |  |  |  |  |  |
|  | Df | SumsOfSqs | MeanSqs | F.Model | R2 | Pr(>F) |  |
| depth | 2 | 0.6659 | 0.33294 | 2.4919 | 0.15376 | 0.001 | *** |
| wetland | 2 | 0.8309 | 0.41546 | 3.1096 | 0.19187 | 0.001 | *** |
| depth:wetland | 4 | 0.5625 | 0.14063 | 1.0526 | 0.1299 | 0.339 |  |
| Residuals | 17 | 2.2713 | 0.13361 | 0.52447 |  |  |  |
| Total | 25 | 4.3306 | 1 |  |  |  |  |
|  |  |  |  |  |  |  |  |
| **LE** |  |  |  |  |  |  |  |
| *envfit* correlations |  |  |  |  |  |  |  |
|  | NMDS1 | NMDS2 | r2 | Pr(>r) |  |  |  |
| depth | -0.00854 | -0.99996 | 0.1857 | 0.397 |  |  |  |
| P | 0.83529 | 0.54981 | 0.2487 | 0.259 |  |  |  |
| S | -0.75718 | 0.65321 | 0.6572 | 0.006 | ** |  |  |
| NO3 | 0.49159 | 0.87083 | 0.1583 | 0.469 |  |  |  |
| CNrat | -0.55046 | 0.83486 | 0.1704 | 0.42 |  |  |  |
| NUTR | 0.81694 | -0.57672 | 0.6571 | 0.005 | ** |  |  |
|  |  |  |  |  |  |  |  |
| perMANOVA |  |  |  |  |  |  |  |
|  | Df | SumsOfSqs | MeanSqs | F.Model | R2 | Pr(>F) |  |
| depth | 2 | 0.22374 | 0.11187 | 0.87484 | 0.1428 | 0.653 |  |
| wetland | 1 | 0.36985 | 0.36985 | 2.89222 | 0.23605 | 0.004 | ** |
| depth:wetland | 2 | 0.20596 | 0.10298 | 0.8053 | 0.13145 | 0.778 |  |
| Residuals | 6 | 0.76726 | 0.12788 | 0.4897 |  |  |  |
| Total | 11 | 1.56681 | 1 |  |  |  |  |
|  |  |  |  |  |  |  |  |
| **NSB** |  |  |  |  |  |  |  |
| *envfit* correlations |  |  |  |  |  |  |  |
|  | NMDS1 | NMDS2 | r2 | Pr(>r) |  |  |  |
| depth | 0.90216 | 0.43141 | 0.3524 | 0.071 | . |  |  |
| P | -0.02069 | -0.99979 | 0.335 | 0.087 | . |  |  |
| S | -0.95822 | -0.28603 | 0.2177 | 0.204 |  |  |  |
| NO3 | 0.25117 | 0.96794 | 0.1095 | 0.492 |  |  |  |
| CNrat | 0.08221 | -0.99662 | 0.5825 | 0.007 | ** |  |  |
| NUTR | -0.46248 | 0.88663 | 0.231 | 0.211 |  |  |  |
|  |  |  |  |  |  |  |  |
| perMANOVA |  |  |  |  |  |  |  |
|  | Df | SumsOfSqs | MeanSqs | F.Model | R2 | Pr(>F) |  |
| depth | 2 | 0.3749 | 0.18745 | 1.24743 | 0.16106 | 0.093 | . |
| wetland | 1 | 0.32491 | 0.32491 | 2.1622 | 0.13958 | 0.003 | ** |
| depth:wetland | 2 | 0.27548 | 0.13774 | 0.91664 | 0.11835 | 0.609 |  |
| Residuals | 9 | 1.35241 | 0.15027 | 0.58101 |  |  |  |
| Total | 14 | 2.32769 | 1 |  |  |  |  |
|  |  |  |  |  |  |  |  |
| **WSB** |  |  |  |  |  |  |  |
| *envfit* correlations |  |  |  |  |  |  |  |
|  | NMDS1 | NMDS2 | r2 | Pr(>r) |  |  |  |
| depth | 0.99887 | 0.04758 | 0.6631 | 0.007 | ** |  |  |
| P | -0.70636 | 0.70785 | 0.4208 | 0.082 | . |  |  |
| S | 0.07951 | -0.99683 | 0.3565 | 0.123 |  |  |  |
| NO3 | -0.81274 | -0.58263 | 0.3583 | 0.115 |  |  |  |
| CNrat | 0.92729 | -0.37435 | 0.6591 | 0.008 | ** |  |  |
| NUTR | -0.64725 | -0.76228 | 0.2799 | 0.179 |  |  |  |
|  |  |  |  |  |  |  |  |
| perMANOVA |  |  |  |  |  |  |  |
|  | Df | SumsOfSqs | MeanSqs | F.Model | R2 | Pr(>F) |  |
| depth | 2 | 0.49774 | 0.24887 | 1.52993 | 0.25902 | 0.014 | * |
| wetland | 1 | 0.19913 | 0.19913 | 1.22418 | 0.10363 | 0.173 |  |
| depth:wetland | 2 | 0.24872 | 0.12436 | 0.76451 | 0.12943 | 0.949 |  |
| Residuals | 6 | 0.976 | 0.16267 | 0.50791 |  |  |  |
| Total | 11 | 1.92159 | 1 |  |  |  |  |
