# Supplementary figures and images for: Microbial community structure and microbial networks correspond to nutrient gradients within coastal wetlands of the Laurentian Great Lakes
Source: FEMS Microbiol Ecol. 2019 Mar 11;95(4):fiz033. doi: 10.1093/femsec/fiz033 (PMC6447756; doi:10.1093/femsec/fiz033)

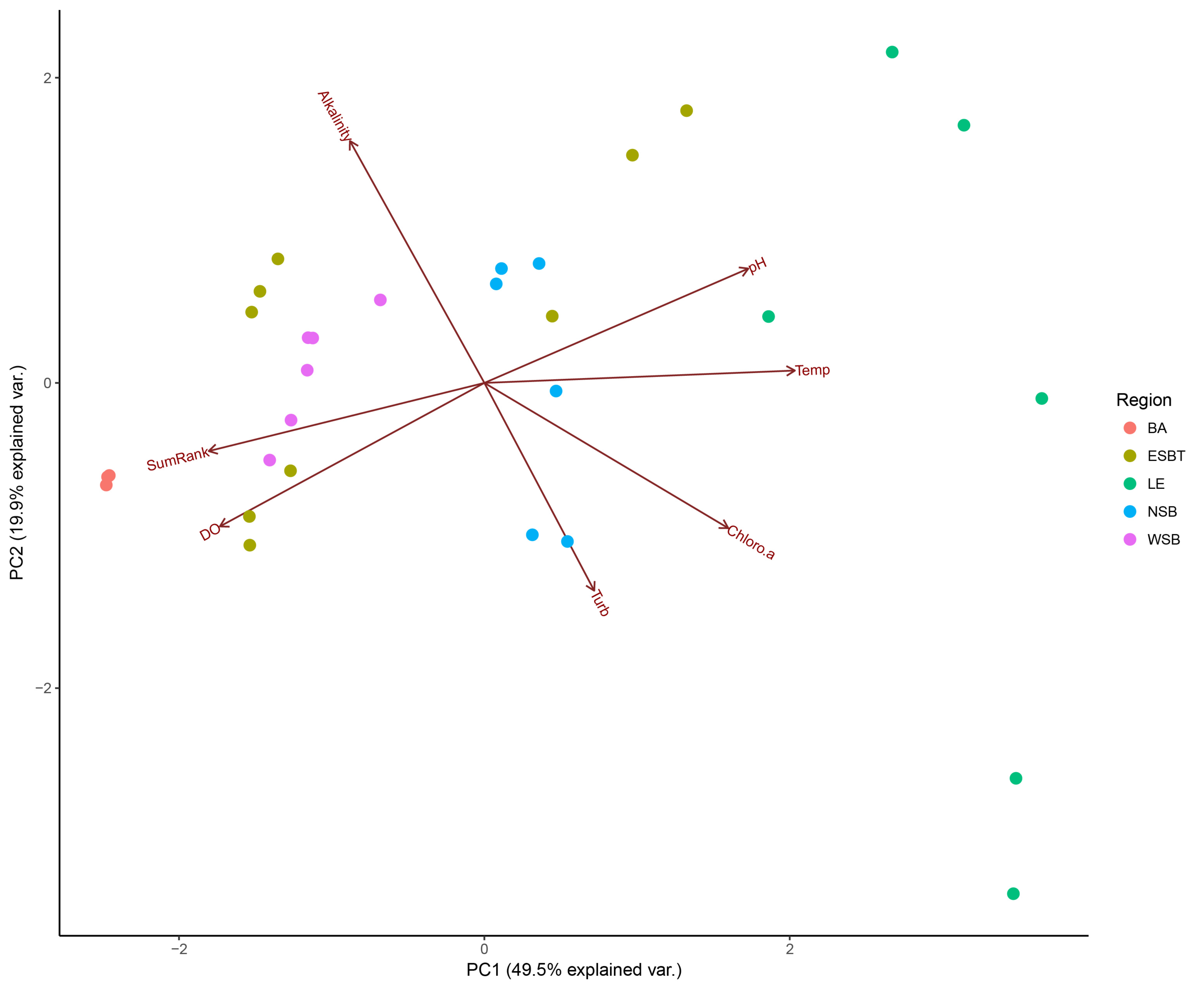

Supplement: Supplemental Files [file fiz033_supplemental_files.zip › Supp_Fig_1._Site_PCA.tif]

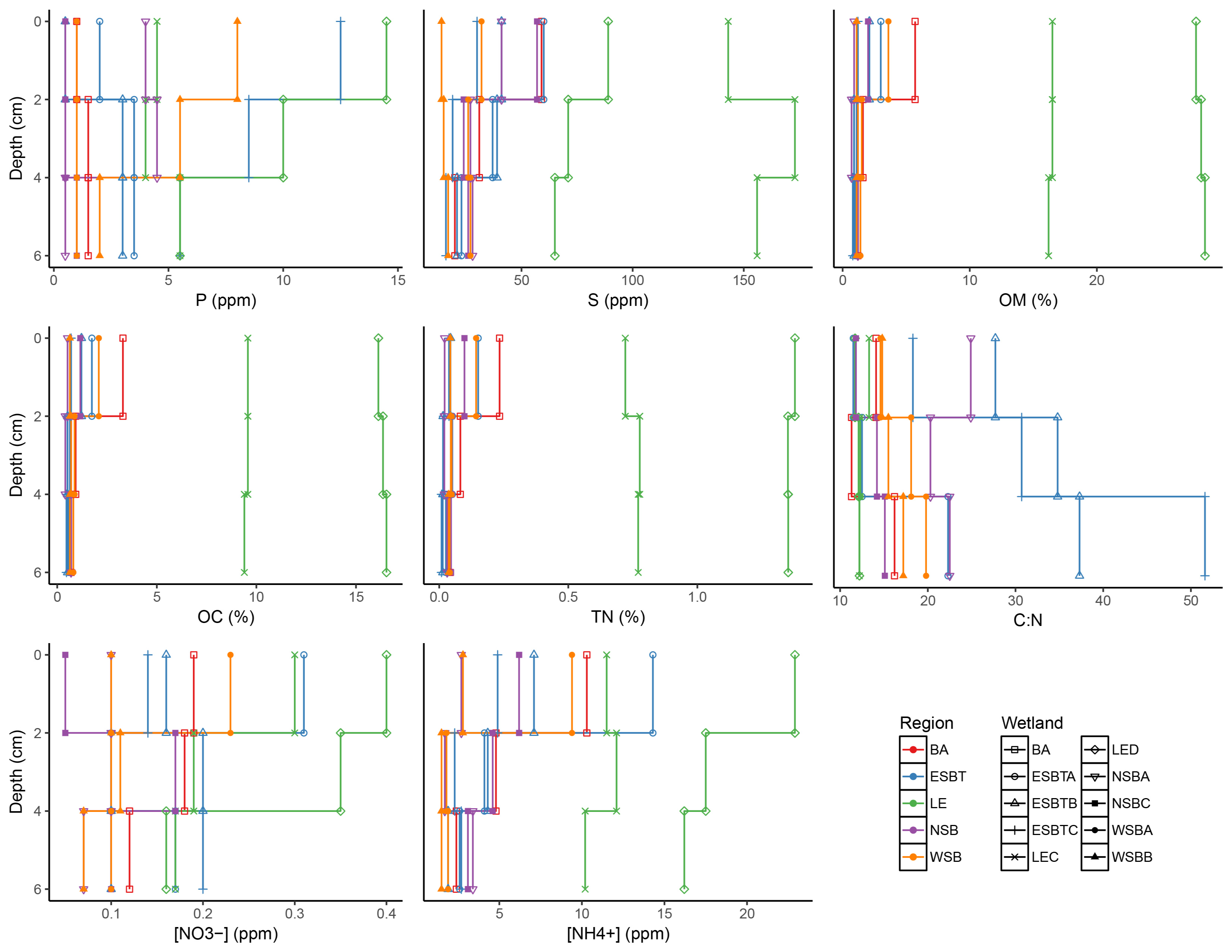

Supplement: Supplemental Files [file fiz033_supplemental_files.zip › Supp_Fig_2._GL_chem_depth_plot.tif]

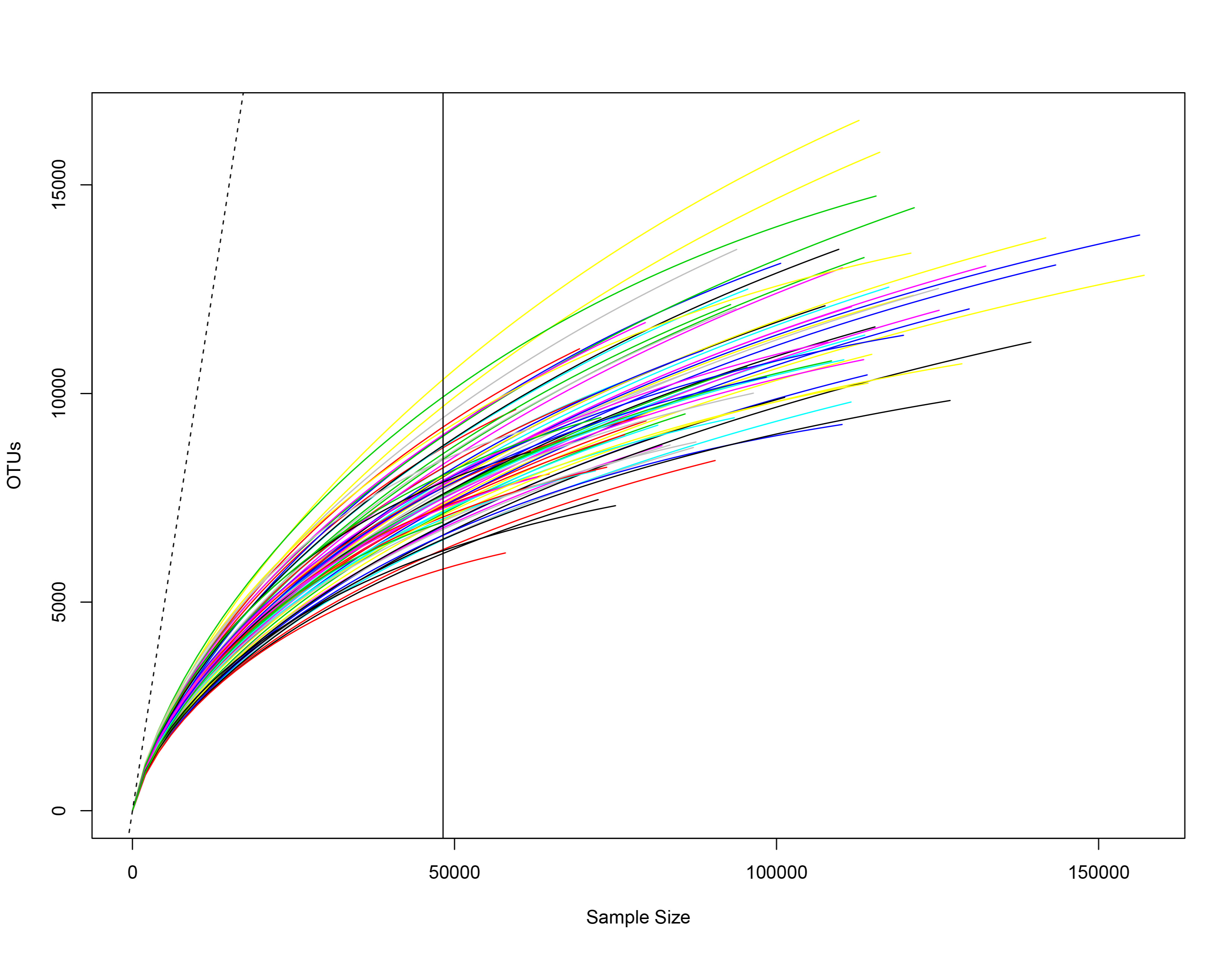

Supplement: Supplemental Files [file fiz033_supplemental_files.zip › Supp_Fig_3._Rarefaction_curve_alpha_div.tif]

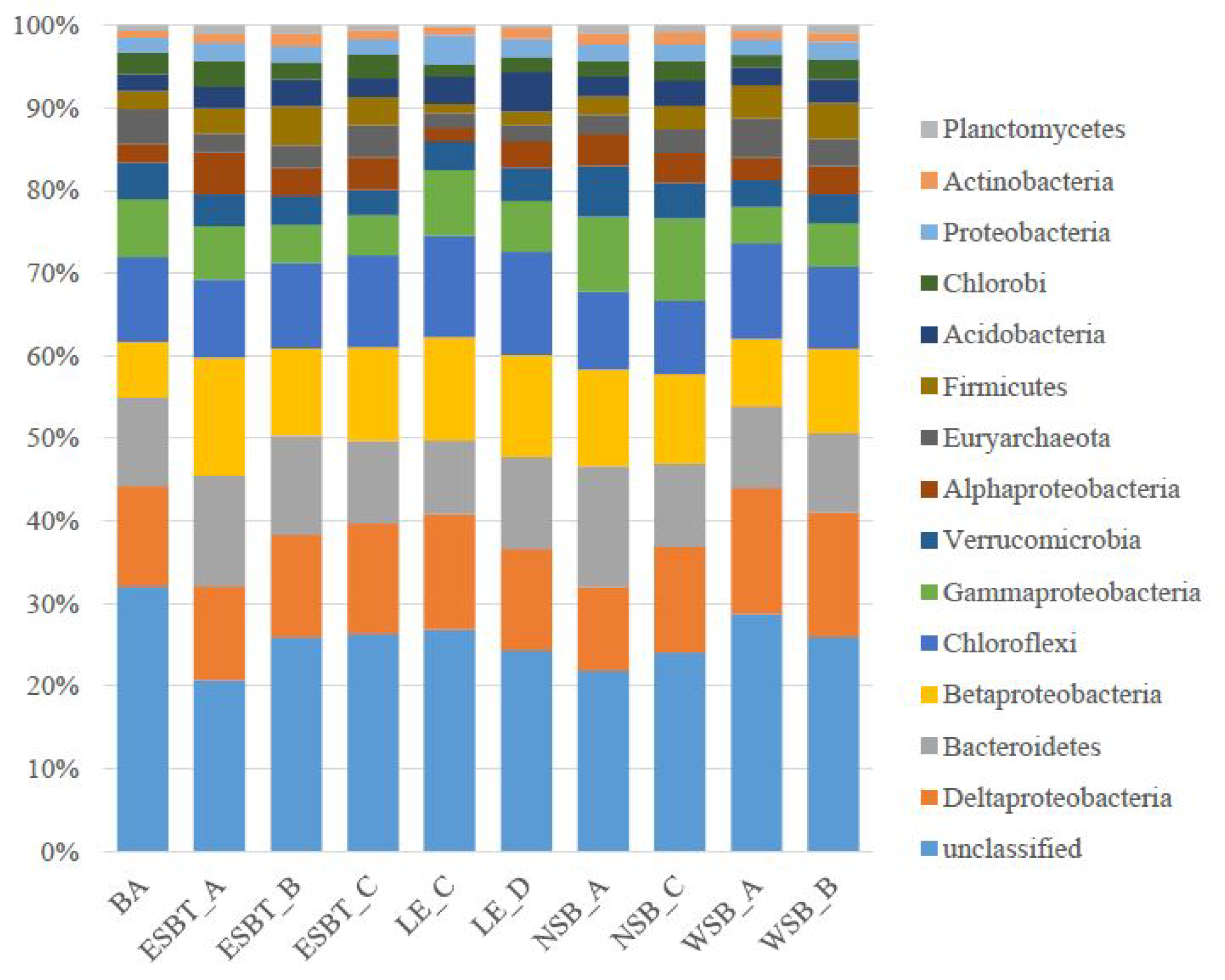

Supplement: Supplemental Files [file fiz033_supplemental_files.zip › Supp_Fig_4._Phylum_breakdown.tif]

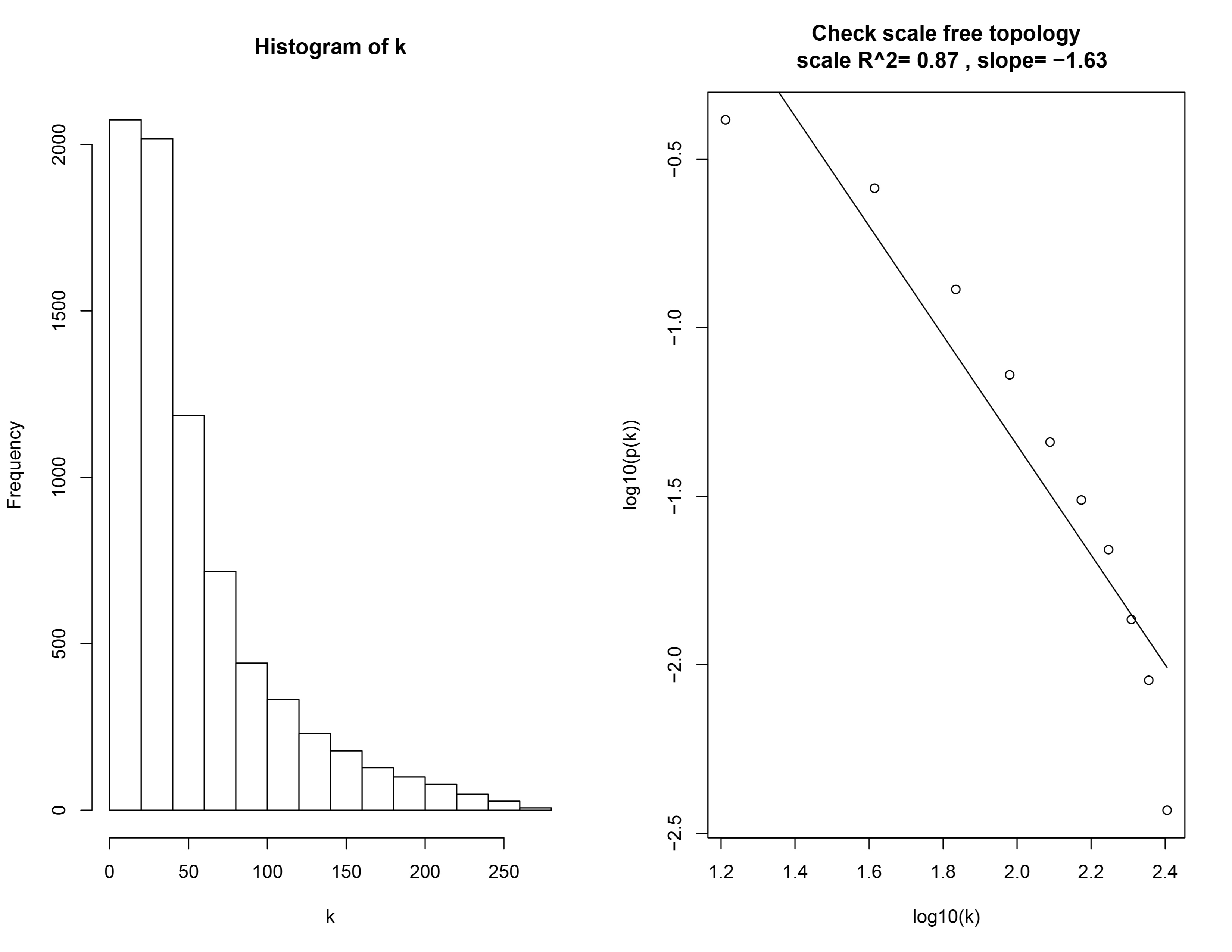

Supplement: Supplemental Files [file fiz033_supplemental_files.zip › Supp_Fig_5._Scale_Free_Plot.tif]

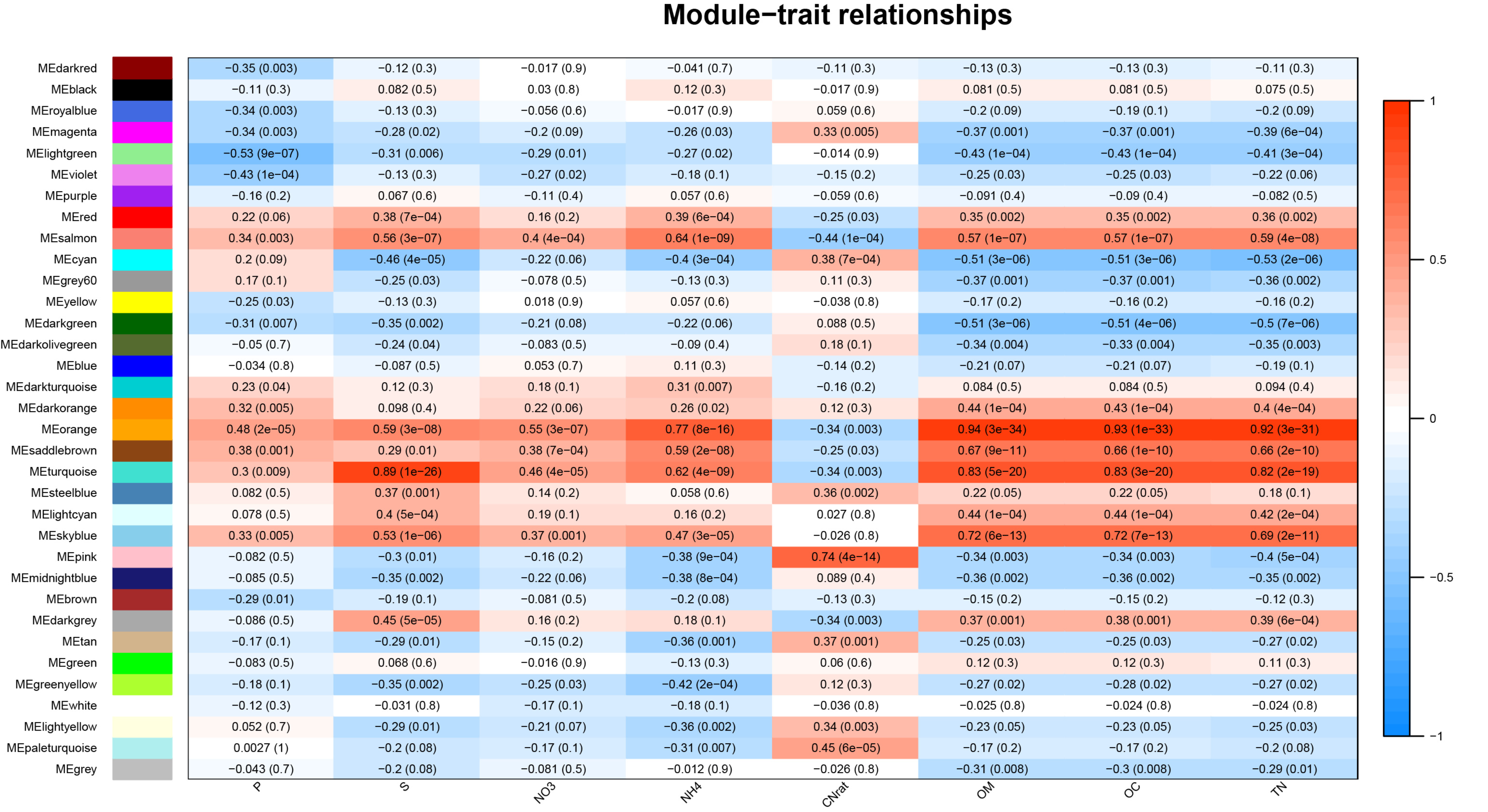

Supplement: Supplemental Files [file fiz033_supplemental_files.zip › Supp_Fig_6._Correlation_WGCNA_table.tif]

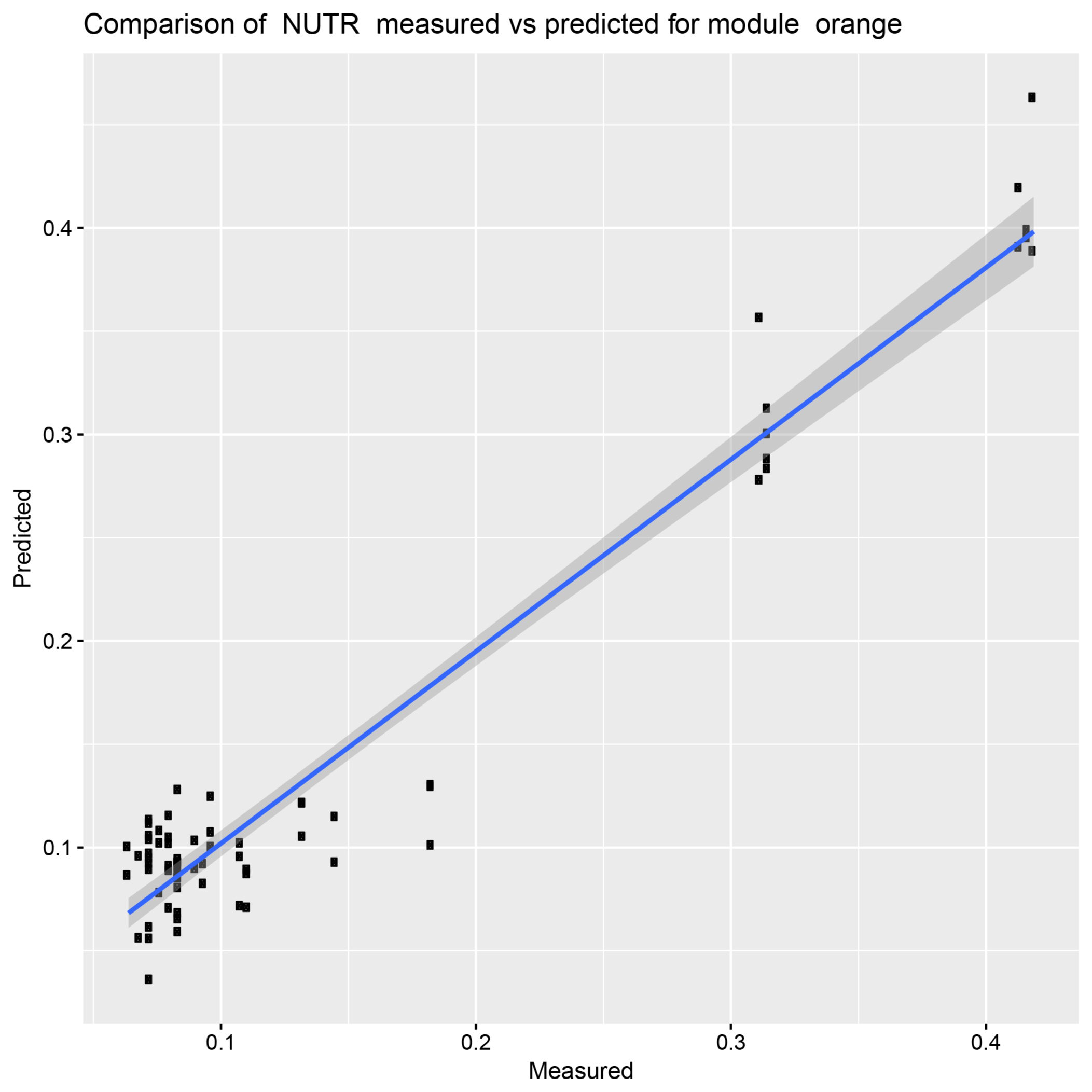

Supplement: Supplemental Files [file fiz033_supplemental_files.zip › Supp_Fig_7._measured_vs_predicted_orange_vs_NUTR.tif]

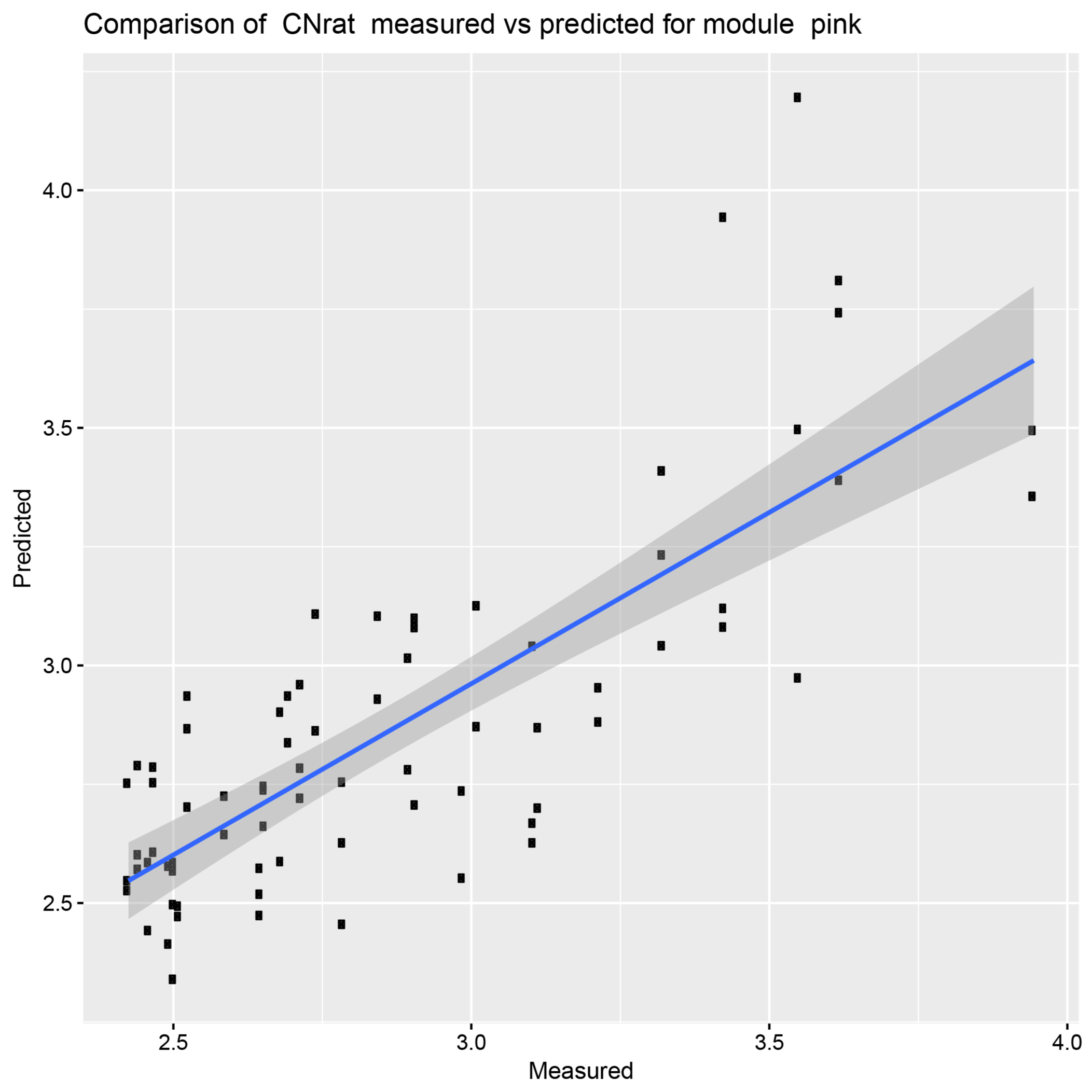

Supplement: Supplemental Files [file fiz033_supplemental_files.zip › Supp_Fig_8._measured_vs_predicted_pink_vs_CNrat.tif]
